# Simultaneous Measurement of Local Pulse Wave Velocities in Radial Arteries Using a Soft Sensor Based on the Fiber Bragg Grating Technique

**DOI:** 10.3390/mi15040507

**Published:** 2024-04-08

**Authors:** Jing Wang, Zhukun Wang, Zijun Zhang, Peiyun Li, Han Pan, Yong Ren, Tuo Hou, Chengbo Wang, Chiew-Foong Kwong, Bei Zhang, Sen Yang, Jing Bie

**Affiliations:** 1 Department of Electrical and Electronic Engineering, University of Nottingham Ningbo China, Ningbo 315100, China; ssyzw7@alumni.nottingham.edu.cn (Z.W.); zijun.zhang@nottingham.edu.cn (Z.Z.); peiyun.li@nottingham.edu.cn (P.L.); skxhp1@nottingham.edu.cn (H.P.); chengbo.wang@nottingham.edu.cn (C.W.); chiew-foong.kwong@nottingham.edu.cn (C.-F.K.); sen.yang@nottingham.edu.cn (S.Y.); 2 Nottingham Ningbo China Beacons of Excellence Research and Innovation Institute, University of Nottingham Ningbo China, Ningbo 315048, China; 3 Key Laboratory of More Electric Aircraft Technology of Zhejiang Province, University of Nottingham Ningbo China, Ningbo 315100, China; 4 Department of Mechanics, Materials and Manufacturing Engineering, University of Nottingham Ningbo China, Ningbo 315100, China; tuo.hou@nottingham.edu.cn; 5 Key Laboratory of Carbonaceous Wastes Processing and Process Intensification Research of Zhejiang Province, University of Nottingham Ningbo China, Ningbo 315100, China; 6 Department of Automation Science and Electrical Engineering, Beihang University, No. 37 Xueyuan Road, Haidian District, Beijing 100191, China; bei.zhang@buaa.edu.cn; 7 Department of Civil Engineering, University of Nottingham Ningbo China, Ningbo 315100, China; jing.bie@nottingham.edu.cn

**Keywords:** fiber Bragg grating, pulse wave velocity, radial artery, cardiovascular disease, wearable sensor, soft sensor, arterial stiffness

## Abstract

Arterial stiffness has been proved to be an important parameter in the evaluation of cardiovascular diseases, and Pulse Wave Velocity (PWV) is a strong indicator of arterial stiffness. Compared to regional PWV (PWV among different arteries), local PWV (PWV within a single artery) outstands in providing higher precision in indicating arterial properties, as regional PWVs are highly affected by multiple parameters, e.g., variations in blood vessel lengths due to individual differences, and multiple reflection effects on the pulse waveform. However, local PWV is less-developed due to its high dependency on the temporal resolution in synchronized signals with usually low signal-to-noise ratios. This paper presents a method for the noninvasive simultaneous measurement of two local PWVs in both left and right radial arteries based on the Fiber Bragg Grating (FBG) technique via correlation analysis of the pulse pairs at the fossa cubitalis and at the wrist. Based on the measurements of five male volunteers at the ages of 19 to 21 years old, the average left radial PWV ranged from 9.44 m/s to 12.35 m/s and the average right radial PWV ranged from 11.50 m/s to 14.83 m/s. What is worth mentioning is that a stable difference between the left and right radial PWVs was observed for each volunteer, ranging from 2.27 m/s to 3.04 m/s. This method enables the dynamic analysis of local PWVs and analysis of their features among different arteries, which will benefit the diagnosis of early-stage arterial stiffening and may bring more insights into the diagnosis of cardiovascular diseases.

## 1. Introduction

Cardiovascular diseases contribute to considerable mortality in modern society [1]. It has been proven that the risk of cardiovascular diseases is related to the degree of arterial stiffness [2]. Arterial stiffening can cause a rise in aortic systolic pressure, a fall in diastolic pressure, and a higher risk of heart diseases with increased age [2,3,4,5,6,7]. In order to identify early-stage arterial diseases so as to perform early intervention, there is an increasing need to monitor arterial stiffness in a convenient, reliable, and noninvasive way.

Pulse Wave Velocity (PWV), the propagation velocity of blood waves/pulses via the circulatory system, is a strong indicator of arterial stiffness [8,9,10,11] and can be used for the prediction of cardiovascular events [12]. The magnitude of PWV highly depends on the property of the blood vessels, e.g., blood vessel elasticity and wall thickness [13]. A common approach used to measure the PWV is to measure the pulses at two arterial sites simultaneously [8,14], and the majority of the existing research based on two-site measurements investigated regional PWVs, i.e., the PWV between two sites located in different arteries, e.g., the brachial–ankle PWV (ba-PWV), carotid–femoral PWV (cf-PWV), and carotid–radial PWV (cr-PWV) [15,16,17], because the long separation between the two sites guarantees a larger scale of the Pulse Transit Time (PTT) and consequently lower requirements for the temporal synchronization of the measurement signals. The research on regional PWVs started in 1922 [18]; in 2013, the cf-PWV was confirmed to be the “gold standard” of assessing arterial stiffness, and it was found that the existence of asymptomatic cardiovascular damage could be confirmed if the cf-PWV is higher than 10 m/s [19]. Nowadays, ba-PWV and cf-PWV have been widely used in clinical applications.

Recently, more research on PWV within a single artery, i.e., local PWV, has been published [20,21,22,23]. Comparing to regional PWVs, there are some significant advantages of local PWVs, as shown in Table 1, especially the capability of identifying minor vascular structural dynamics which are usually suppressed by regional PWVs.

Various types of signals have been adopted for the measurement of local PWVs, such as the arterial pressure wave [27], photoplethysmograph (PPG) [26], magnetic plethysmograph (MPG) [28], bioimpedance plethysmograph (IPG) [29], cardiovascular magnetic resonance imaging [30], and ultrasound imaging [31] techniques; however, the local PWVs measured through these methods vary significantly in their scale [32], which could be due to the following factors:

1. Variation in signal quality. For the signals used by local PWVs, which are usually obtained in a noninvasive way, the signal-to-noise ratio (SNR) is usually not high enough, thus induces low accuracy and low reproducibility.
2. Inconsistent selection of fiducial points in the pulses’ time domain for the calculation of local PWVs. The pulse waveform feature is to some extent affected by the wave reflection effect, which increases the error induced by the selection of fiducial points for different pulse measurement sites.
3. Data/signal processing techniques. Some data/signal processing techniques, either in hardware or software, e.g., filtering, smoothing, or averaging, impose changes to the pulse feature in the time domain, thus inducing errors in the calculation of local PWVs and/or suppressed PWV dynamic features [33,34,35].

The measurement of pulses has mainly been conducted with electronic sensors, because of the mature development of these techniques in hardware [36]. However, Fiber Bragg Grating (FBG) has been more frequently used to monitor pulses in recent years, considering its unique advantages, e.g., its high sensitivity, immunity to electromagnetic interference, light weight, skin-friendly properties, low cost, and high flexibility. By now, FBG has been used to measure pulses noninvasively for different applications based on pulse waveform feature analysis [37,38,39], for example in cardiorespiratory [40], blood pressure [41], and arterial compliance [42]. So far, there has been only one study, published in 2015, that measured the carotid–radial PTT [43] using FBG for the analysis of systolic blood pressure. In that paper, two separate optical fibers each inscribed with an FBG unit were used to measure pulses at the wrist and at the carotid arterial sites; however, the cr-PWV presented in this paper was 0.45 m/s [43], which is far less than the cr-PWV scale found through other methods [44]. There has been no research published on the simultaneous measurement of more than one local PWV so far.

This paper presents a method for the simultaneous measurement of local PWVs in radial arteries based on the FBG technique. The pulses at the wrists and at the fossa cubitalis in each radial artery were measured simultaneously using two optical fibers, each of which was inscribed with two FBG units. Compared to the existing state-of-the-art local PWV methods, the method presented in this paper has the following advantages:

1. Simultaneous measurement of two local PWVs in two radial arteries without synchronization challenges. The optical signals at each pulsation site were measured simultaneously, as they shared the same optical path and were measured with the FBG Interrogator at the same time. The technical challenges in synchronizing electronic signals, e.g., the time delay due to transmission line theory, do not exist. Therefore, this method provides the possibility for investigation of the features of local PWVs within multiple main arteries simultaneously.
2. With a moderate level of SNR (3.7 ± 0.6), and without altering the waveform features, this method provides local PWVs with an error of about 3.52%, leading to an improvement in the accuracy and precision of the measurement.
3. The local PWV given by this method is independent from the wave reflection effect because it uses a segment of raw data rather than a single fiducial point.
4. This method allows the investigation of the dynamic features of local PWVs in a single radial artery and the investigation of local PWV differences in two radial arteries.
5. The pulse features in both the time and frequency domains are not altered due to the use of raw data; therefore, the accuracy in the calculation of local PWV is improved.

## 2. Materials and Methods

**Figure 1:**
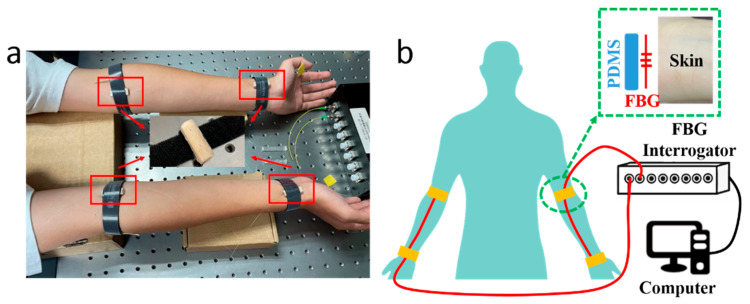
(**a**) A photo of the PWV measurement with the FBG units attached to four pulse sites on a male volunteer, i.e., at the fossa cubitalis and the wrist on both the left and the right arms, where the two pulse sites on the same arm shared the same optical fiber. The volunteer sat still and kept calm during the measurement process. (**b**) A schematic of the PWV measuring system consisting of two optical fibers (red wire), an FBG Interrogator, and a computer. A closer view of the pulse measurement at the pulse site is presented as well, where the FBG unit on the optical fiber was placed in between the PDMS substrate and the skin.

The PWV measuring system consists of 2 optical fibers inscribed with FBG sensors, which were attached to the skin at the pulse sites on radial arteries, an FBG Interrogator (GC-97001C-02-08, Arcadia Optronix Ltd., Zhuhai, China) functioning as both a light source and an optical detector to analyze the reflected spectra from the FBGs, and a computer for data storage, as shown in Figure 1, wherein Figure 1a presents a photo of the PWV measurement on a volunteer and Figure 1b presents the schematic of the system proposed in this paper.

Semi-cylindrical polydimethylsiloxane (PDMS) was prepared as the substrate for the FBGs. The PDMS (SYLGARD™ 184 Silicone Elastomer Base, Dow, Midland, MI, USA) and the curing agent (SYLGARD™ 184 Silicone Elastomer Curing Agent, Dow, USA) were mixed with a mass ratio of 10:1, then stirred for 10 min. The solution was injected into a cylindrical mold; then, the whole set was placed into an oven (DHG-9000(A)), which was preheated to 80 °C, for 1 h curing. Afterwards, the whole set was taken out to cool down to room temperature, and was cut in half to form semi-cylindrical PDMS substrates. The FBG unit on the optical fiber was placed between the selected pulse sites on the arms and the cylindrical side of the PDMS substrate, then fastened with a Velcro strap, a closer view of which is presented in Figure 1b. The fiber input was connected to the FBG Interrogator, and the fiber output was enclosed with an optical terminator. The FBG Interrogator worked at a sampling frequency of 1 kHz with a Least Significant Bit (LSB) of 1 pm. The separation between FBGs at the fossa cubitalis and at the wrist was measured manually. A demonstration video was provided as Supplementary Material S1 with this paper.

## 3. Results and Discussions

### 3.1. Signal Preprocessing Analysis

**Figure 2:**
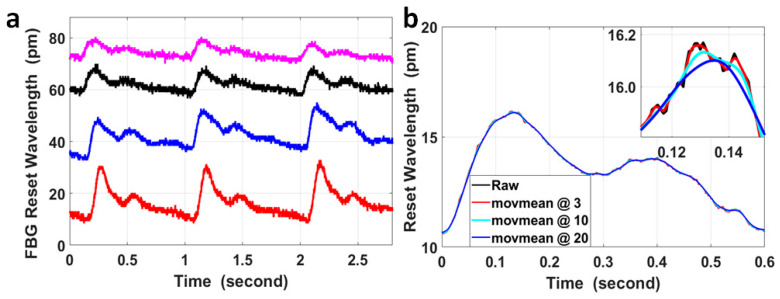
(**a**) An example of the raw experimental data of FBG wavelengths measured at the wrist and at the fossa cubitalis on the left and the right arms of volunteer No. 1. Corresponding to the four curves from top to bottom, the pulse sites were the right fossa cubitalis, right wrist, left fossa cubitalis, and left wrist, respectively. The four curves are presented with different offsets in wavelengths for the purpose of better data presentation, which is irrelevant to the PTT or PWV calculation. (**b**) A comparison of the raw data of a single pulse (black curve) with the curves after moving average with different sizes of windows of the sample numbers, wherein moving average for the red curve was with a parameter of 3, for the light blue curve with a parameter of 10, and for the blue curve with a parameter of 20. The inset figure shows the magnified features around the pulse peak within a time window of 40 ms.

The wavelength responses of the four FBGs attached to the wrist and the fossa cubitalis on both arms of volunteer No. 1 were collected using the FBG Interrogator, and examples of the raw data at the four pulse sites are presented in Figure 2a. The four curves are presented with different offsets in wavelength only for the purpose of better data presentation, which is not included in the signal processing method used in this paper. The pulse-induced wavelength shifts at the four sites varied in amplitude, which was mainly caused by three reasons, i.e., the variation in the pulse intensity, the variation in the blood vessel depth from the skin, and the variation in the tightness of the fixing straps holding the FBG onto the skin. The average signal-to-noise ratio (SNR) of the four measurements is 3.7 ± 0.6.

The FBG wavelength shift induced by pulses was within the range of 6.4 pm to 22.1 pm for the measurements presented in Figure 2a. The resolution in wavelength of the FBG Interrogator used in the sensing system was 1 pm, so the quantization error generated by the device was noticeable in the signal (the small spikes on the black curve in Figure 2a) and could not be neglected. Moving the average with different window sizes was used to process the signal in order to analyze its effect on the temporal features of the pulses, and these results are presented in Figure 2b.

The curves after moving the average with different window sizes displayed quite similar features with the raw signal on the time scale of 0.6 s, but on a time scale of 40 ms, the curves after moving the average with parameters of 10 and 20 were much smoother compared with using a parameter of 3; however, the peak of these curves was shifted with a time delay of 2 ms and 6 ms, respectively, compared to the peak of the curve after moving the average with a parameter of 3. Assuming the blood vessel length between the pulse sites at the wrist and at the fossa cubitalis was 30 cm and the local PWV within this radial artery segment was 14 m/s (a median value based on currently published results), the corresponding PTT would be 21.4 ms. Under the worst circumstance, the delay of 6 ms for each pulse and the overall delay of up to ±6 ms for both pulses would significantly impact the PTT and local PWV amplitudes. On the other hand, even though it was possible that the time delay induced by moving the average on the corresponding pulse pair was similar, considering the small scale of the PTT for local PWV, it would be preferred to avoid any false time delay as much as possible in order to improve the PTT accuracy. So, in order to preserve the original temporal features of the pulses, especially for low-SNR pulse signals, the raw data were used for calculation of the PTT without any signal preprocessing.

### 3.2. Local PWV in Radial Arteries

By nature, PWV is a parameter determined by the blood vessel properties, which can be calculated using the Moens–Korteweg equation (Equation (1)), where E is the elastic modulus of the blood vessel; g is the gravitational constant; a is the wall thickness; ρ is the blood density within the measured vessel; and d is the interior diameter of the vessel [45].

(1) $$\mathrm{PWV}=\sqrt{\frac{Ega}{\rho d}}$$

For PWV measured based on two-site methods, this can be calculated using Equation (2), where *K* is the length of the blood vessel between the two measuring sites, and PTT is the time interval for a single pulse wave to travel between the two measuring sites [46]. Equation (2) was used in this paper for local PWV calculation.

(2) $$\mathrm{PWV}=\frac{K}{\mathrm{PTT}}$$

Regarding the value of *K* in Equation (2), the experience equation for blood vessel length calculation based on height [24] is not applicable for a radial artery segment between the fossa cubitalis and wrist. Since the separation between the wrist and the fossa cubitalis is relatively small, the direct distance between the FBGs attached to both sites was measured and used as *K* in Equation (2). The actual length of the radial artery segment shall be longer than the *K* value we used; however, in terms of the amplitude of the calculated local PWV, the variation in *K* only “inflates” or “deflates” its amplitude at the same proportion for all local PWVs and for all volunteers. Its effect can thus be ignored, especially when the difference in local PWVs is discussed.

**Figure 3:**
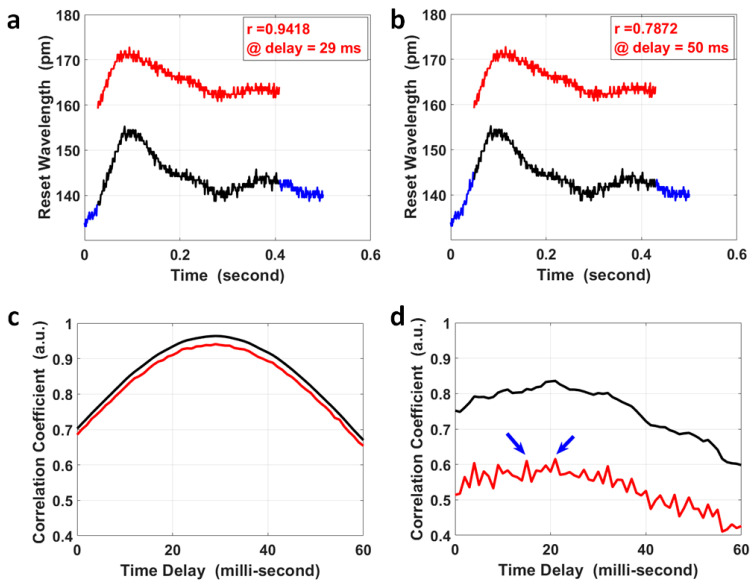
The calculation of PTT via the correlation analysis of pulses at the wrist and at the fossa cubitalis. The pulse segment at the fossa cubitalis, the red curve in (**a**,**b**), was compared with the pulse segment at the wrist, the black curve in (**a**,**b**), wherein the wrist pulse segment shifted within its own pulse duration (the blue curve). The correlation coefficient of the pulse segments at the two sites was 0.9418 at a time delay of 29 ms (**a**), and 0.7872 at a time delay of 50 ms (**b**), respectively. (**c**) The correlation coefficient of the two corresponding pulse segments at the wrist and at the fossa cubitalis at different time delays, wherein the red curve shows the results using raw experimental data and the black curve is based on data after moving the average with a parameter of 3. (**d**) The correlation coefficient of two corresponding pulse pairs with an ultra-low SNR, using raw experimental data (red curve) and using data after moving the average with a parameter of 3 (black curve). There are two peaks in the red curve (indicated by the blue arrows) presents similar amplitude, which could be determined based on the black curve at the same time delay.

The waveform features of pulses measured at the wrist and at the fossa cubitalis are quite similar to the minimum effect of multiple reflection; meanwhile, the pulses at both sites were measured simultaneously. Therefore, the correlation coefficient is an ideal parameter for their time delay calculation, which is PTT in Equation (2). Equation (3) was used for calculating the correlation coefficient between the two pulses at the wrist (*X* in Equation (3)) and at the fossa cubitalis (*Y* in Equation (3)):

(3) $$r=\frac{Cov\left(X,Y\right)}{\sqrt{Var\left[X\right]Var\left[Y\right]}}$$

where $Cov\left(X,Y\right)$ is the covariance of variables *X* and *Y*; $Var\left[X\right]$ is the variance of variable *X*; and $Var\left[Y\right]$ is the variance of the variable *Y*. A segment of the pulse at the fossa cubitalis (*Y*) was selected that covered the two peaks in a single pulse, shown as the red curve in Figure 3a,b; the same length of segment for its corresponding pulse at the wrist (*X*) was selected to calculate their correlation coefficients at various time delays (black curve in Figure 3a,b), and the overall results are presented as the red curve in Figure 3c. The correlation coefficient achieved a maximum value of 0.942 when the time delay was 29 ms, which was the PTT for the pulse to propagate from the fossa cubitalis to the wrist. As a comparison, the correlation coefficient at various time delays was also calculated for the same pair of pulses after moving the average with a parameter of 3, i.e., the black curve in Figure 3c, and the maximum coefficient was 0.964 at the same time delay with an improvement in the correlation coefficient of only 2.34%. Even though it obviously affected the pulse amplitude, the quantization error imposed much less effect on the correlation coefficient. The reason for this was that the pulse waves at the fossa cubitalis and at the wrist shared the same optical fiber and the same channel on the FBG Interrogator, so the noises induced by the equipment, the environment, and the participant were the same for both pulses on the same artery, thus would not affect the correlation coefficient calculation. This proves the reliability of this method in the calculation of PTT based on the correlation coefficient using raw data even with a low SNR, with the precondition that both pulses are measured simultaneously.

For special cases where the SNR of the pulse pair is ultra-low (2 pairs out of 383 pairs in our measurement), the correlation coefficient results using raw pulse data might be more noisy, as the red curve shows in Figure 3d, where there were two peaks with similar correlation coefficient values. The possible error in PTT calculation for these special cases could be avoided by setting a correlation coefficient threshold using raw data (e.g., 80%). When the raw data delivers a maximum correlation coefficient less than the threshold, the correlation coefficient using pulses after moving the average with a parameter of 3 could be used to calculate the PTT, as the black curve shows in Figure 3d, to serve as a reference PTT for the results using raw data by narrowing down the time window of interest.

The precision in PTT calculation was mainly determined by the sampling rate, i.e., 1 kHz; so, the PTT in Figure 3c was 29 ± 1 ms. The separation between the two pulse sites on the left forearm was 268 ± 0.2 mm; so, the PWV within the left radial artery could be calculated together with its error determined by both the error in PTT and the error in separation measurement. The local PWV was 9.25 ± 0.33 m/s, with an error of about 3.52%. This local PWV was within the same range of the ba-PWV for healthy adults at the same age of 21 years old. What is worth mentioning is that the separation between the two pulse sites for local PWV calculation was their separation measured on the skin, different from the separation for regional PWV calculated using the experience equation based on human height.

### 3.3. Analysis of General Local PWVs

For PWV in the radial artery, assuming that the blood vessel length between the pulse sites at the wrist and at the fossa cubitalis was 30 cm and that the local PWV range was from 6 m/s to 20 m/s, the PTT ranged from 15 ms to 50 ms, which is far less than the pulse period ranging from 500 ms to 1200 ms for a heart rate ranging from 50 bpm to 120 bpm. Therefore, the PTT from the fossa cubitalis to the wrist would be within one pulse delay, and the PTT for other local PWVs and even regional PWVs would be within one pulse delay for the majority of cases.

**Figure 4:**
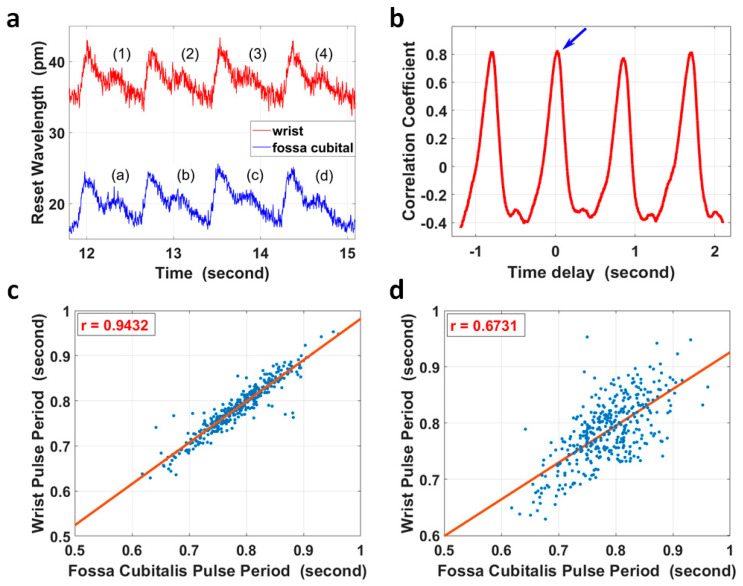
(**a**) The pulse waves within the radial artery of the right forearm at the wrist (red curve) and at the fossa cubitalis (blue curve) of volunteer No. 1, respectively. (**b**) Pulse (2) of the red curve in (**a**) at the wrist was selected and its correlation coefficients with four other pulses of the blue curve at the fossa cubitalis were calculated. The blue arrow shows the correlation coefficient of pulse (2) and pulse (**b**). (**c**) The correlation analysis of 383 pulse periods of corresponding pulse pairs. (**d**) The correlation analysis of the same pulses in (**c**) but with one extra pulse period delay.

However, for extreme cases, for example, with ultra-low local PWV in a relatively longer artery segment, it was possible that the PTT between two pulse sites was larger than one pulse period. In this case, simple correlation analysis of the corresponding/non-corresponding pulse pair could not identify the difference, as the correlation coefficient of the two non-corresponding pulses had a maximum value that was close to that of the corresponding pulse pair. Taking the four pulse pairs in Figure 4a as an example, the segment from pulse (2) at the wrist in Figure 4a was compared with four pulses at the fossa cubitalis in Figure 4a accordingly, and the correlation coefficient with different time delays is shown in Figure 4b. The blue arrow indicates the maximum correlation coefficient between pulse (2) and pulse (b), which were the corresponding pulse pair; however, pulse (2) also presented a high correlation with pulses (a), (c), and (d), and its correlation with pulse (b) did not show a distinct difference in amplitude.

The heart rate variation [47] characteristic could be used to check if the PTT was within one pulse period for the majority of cases or longer for extreme cases, as shown in Figure 4c,d. The pulse duration of 383 pairs of pulses at the wrist and at the fossa cubitalis were calculated, and they showed a correlation coefficient of 0.943 in pulse duration when the pulses were analyzed in corresponding pairs, and this correlation coefficient reduced to 0.673 when the pulses were analyzed with one extra pulse delay added.

### 3.4. Local PWV Variation in Two Radial Arteries

**Figure 5:**
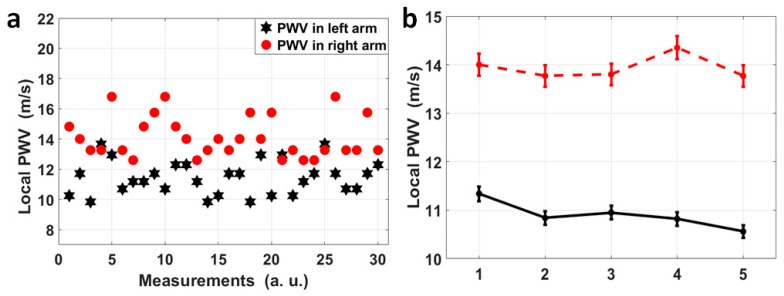
(**a**) A scatter diagram of 30 pairs of local PWVs in the left (black) and right (red) radial arteries for volunteer No. 1. Thirty pairs of pulses were measured continuously and each pair was used to calculated a local PWV, and the variation in PWV across time reflects the heart rate variation, and the separation between the red dots and the black stars can be obviously observed. (**b**) Each dot in this figure represents the average of left (black)/right (red) radial PWVs based on 1 min measurements on volunteer No. 1, and these 1 min measurements on volunteer No. 1 were repeated five times. A stable difference in average radial PWVs between the left and right can be observed.

For volunteer No. 1, who sat still and kept calm during the measurement, the four pulse sites on both radial arteries were measured simultaneously, and for each radial artery, the pulse pairs at the fossa cubitalis and at the wrist were analyzed separately so that the dynamic features of the local PWVs in a single radial artery could be observed, as shown in Figure 5a. The black stars indicate the local PWVs calculated using 30 pulse pairs on the left radial artery, while the red dots represent local PWVs on the right radial artery. It can be observed that the local PWVs varied in amplitude, i.e., the right radial PWV varied from 12.60 m/s to 16.80 m/s and the left radial PWV varied from 9.84 m/s to 13.67 m/s. Even though there is overlap in their ranges, 27 out of 30 right radial PWVs were larger than the left radial PWVs, and the average right radial PWV (14.12 m/s) was also larger than the average left radial PWV (11.44 m/s). Five more measurements were taken, with each measurement having a 1 min duration, for the same volunteer who kept the same status, and the average and the standard deviation of the left and right radial PWVs for each measurement are shown in Figure 5b. This showed a stable state of the larger right radial PWV compared to the left, with a difference of 3.04 m/s averaged out of five measurements.

**Figure 6:**
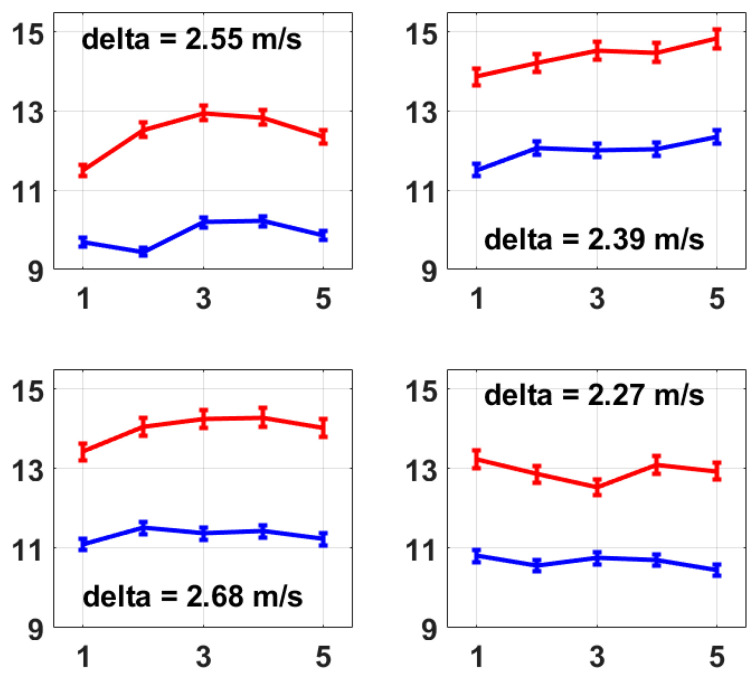
These four figures present the average left and right radial PWVs from volunteer No. 2 to volunteer No. 5. The calculation of average radial PWV was the same as that described for Figure 5b, i.e., based on 1 min measurements which were repeated five times. A stable difference in average radial PWV between the left and right is also observed on each volunteer from No. 2 to No. 5, which presents the same conclusion as Figure 5b.

The same measurements were taken on four other volunteers (herein referred to as No. 2 to No. 5), and their average left and right radial PWVs are shown in Figure 6. The raw experimental data and the processing program were provided as Supplementary Material S2 and S3, respectively. The results all show a stable difference between the left and right radial PWVs within the range of 2.00 m/s to 3.00 m/s on average. The five volunteers were male, aged 19 to 21 years old, and physically fit and healthy, and based on their measurements using this method, the average left radial PWV ranged from 9.44 m/s to 12.35 m/s and the average right radial PWV ranged from 11.50 m/s to 14.83 m/s, and the average right radial PWV was larger than the average left radial PWV with a difference ranging from 2.27 m/s to 3.04 m/s. A possible reason for the noticeably larger right radial PWVs could be that the hardness of the right radial artery was increased due to the more frequent usage of the right arm as all five volunteers were right-handed, but this would need future work to be confirmed. However, as a proof of concept, this method presented here provides a possible and reliable solution for PWV feature analysis within the main arteries for both healthy people and people with cardiovascular diseases, showing that the simultaneous measurement of multiple PWVs is possible to bring more abundant information to clinical applications.

## 4. Conclusions

This paper presents a method for the simultaneous measurement of two local radial PWVs based on the Fiber Bragg Grating technique, wherein correlation analysis of the corresponding pulses at the fossa cubitalis and at the wrist was used to calculate the PTT and the corresponding local PWVs. Based on the measurements of five male volunteers aged 19 to 21 years old, the average left radial PWV ranged from 9.44 m/s to 12.35 m/s and the average right radial PWV ranged from 11.50 m/s to 14.83 m/s, and for each volunteer, the average right radial PWV was higher than the average left radial PWV with an obvious difference ranging from 2.27 m/s to 3.04 m/s. This method supports the dynamic analysis of local PWV, and supports the analysis of local PWV features within different arteries. This method can be further developed to allow the simultaneous measurement of multiple local PWVs and multiple regional PWVs, so it has high potential in clinical applications as it can provide more detailed and abundant information for the evaluation of blood vessel properties.

## Figures and Tables

**Table 1:** Comparison of features of regional PWV and local PWV.

Features	Regional PWV	Local PWV
Artery of interest	Two different arteries.	Single artery.
Arterial length	Relatively long. For human heights of 150 cm to 200 cm, the arterial length for ba-PWV is from 165.1 cm to 216.7 cm, using the experience equation of blood vessel length calculation based on height [24].	Relatively short: e.g., usually <30 cm for radial local PWV between the fossa cubitalis and wrist. Length is not used for the calculation of local PWV if the measurement is based on an ultrasonic technique [5].
Error in arterial length	Usually large error due to two reasons: 1. Error induced by the experience equation in calculating blood vessel length based on height, as it has been proven that the blood vessel length is significantly longer than the experience-calculated value [25]; 2. Variation in blood vessel length due to individual difference.	Comparatively lower error [26], as the arterial length is of a much smaller scale; thus, the errors induced by the experience equation and by individual vessel variation are considerably lower.
Arterial stiffness	PWV measurement is averaged over different arteries.	Specific measurement within a single artery or an arterial segment.
Pulse waveform feature	Highly affected by the multiple reflected pulse waves between different arteries [13].	Much less affected by reflected pulse waves, especially when the arterial segment is short.
Dynamic features of PWV	Cannot be captured.	Can be captured with some methods.
Clinical application	Widely used, especially ba-PWV and cf-PWV (gold standard).	Not yet. The published local PWV amplitudes obtained through different methods vary in a large range.

## Data Availability

The raw experimental data have been provided as one of the Supplementary Materials.

## Supplementary Materials

The following supporting information can be downloaded at: https://www.mdpi.com/article/10.3390/mi15040507/s1, S1: Demo Video; S2: Raw Data; S3: Program.

## References

[1] Najjar S., Scuteri A., Lakatta E. (2005). Arterial Aging Is It an Immutable Cardiovascular Risk Factor?. Hypertension.

[2] Kang S., Fan H.-M., Li J., Fan L.-Y., Miao A.-Y., Bao Y., Wu L.-Z., Zhu Y., Zhang D.-F., Liu Z.-M. (2010). Relationship of Arterial Stiffness and Early Mild Diastolic Heart Failure in General Middle and Aged Population. Eur. Heart J..

[3] Mattace-Raso F.U.S., van der Cammen T.J.M., Hofman A., van Popele N.M., Bos M.L., Schalekamp M.A.D.H., Asmar R., Reneman R.S., Hoeks A.P.G., Breteler M.M.B. (2006). Arterial Stiffness and Risk of Coronary Heart Disease and Stroke. Circulation.

[4] Laurent S., Katsahian S., Fassot C., Tropeano A.-I., Gautier I., Laloux B., Boutouyrie P. (2003). Aortic Stiffness Is an Independent Predictor of Fatal Stroke in Essential Hypertension. Stroke.

[5] Wang Z., Yang Y., Yuan L.-J., Liu J., Duan Y.-Y., Cao T.-S. (2015). Noninvasive Method for Measuring Local Pulse Wave Velocity by Dual Pulse Wave Doppler: In Vitro and in Vivo Studies. PLoS ONE.

[6] Darwich M.A., Langevin F., Darwich K. (2015). Local Pulse Wave Velocity Estimation in the Carotids Using Dynamic MR Sequences. J. Biomed. Sci. Eng..

[7] Nabeel P.M., Jayaraj J., Srinivasa K., Mohanasankar S., Chenniappan M. (2018). Bi-Modal Arterial Compliance Probe for Calibration-Free Cuffless Blood Pressure Estimation. IEEE Trans. Biomed. Eng..

[8] Lim T.H., Chung S.M., Lee D.S., Choi S.R., Moon J.S., Yoon J.S., Won K.C., Lee H.W. (2020). Peripheral Arterial Stiffness Increases the Risk of Progression of Renal Disease in Type 2 Diabetic Patients. Front. Med..

[9] The Reference Values for Arterial Stiffness’ Collaboration (2010). Determinants of Pulse Wave Velocity in Healthy People and in the Presence of Cardiovascular Risk Factors: ‘Establishing Normal and Reference Values’. Eur. Heart J..

[10] Mackenzie I.S., Wilkinson I.B., Cockcroft J.R. (2002). Assessment of Arterial Stiffness in Clinical Practice. QJM Mon. J. Assoc. Physicians.

[11] Laurent S., Cockcroft J., Van Bortel L., Boutouyrie P., Giannattasio C., Hayoz D., Pannier B., Vlachopoulos C., Wilkinson I., Struijker-Boudier H. (2006). Expert Consensus Document on Arterial Stiffness: Methodological Issues and Clinical Applications. Eur. Heart J..

[12] Ben-Shlomo Y., Spears M., Boustred C., May M., Anderson S.G., Benjamin E.J., Boutouyrie P., Cameron J., Chen C.-H., Cruickshank J.K. (2014). Aortic Pulse Wave Velocity Improves Cardiovascular Event Prediction: An Individual Participant Meta-Analysis of Prospective Observational Data from 17,635 Subjects. J. Am. Coll. Cardiol..

[13] Nabeel P.M., Kiran V.R., Joseph J., Abhidev V.V., Sivaprakasam M. (2020). Local Pulse Wave Velocity: Theory, Methods, Advancements, and Clinical Applications. IEEE Rev. Biomed. Eng..

[14] Hirata K., Kawakami M., O’Rourke M.F. (2006). Pulse Wave Analysis and Pulse Wave Velocity A Review of Blood Pressure Interpretation 100 Years after Korotkov. Circ. J..

[15] Bikia V., Rovas G., Pagoulatou S., Stergiopulos N. (2021). Determination of Aortic Characteristic Impedance and Total Arterial Compliance from Regional Pulse Wave Velocities Using Machine Learning: An in-silico Study. Front. Bioeng. Biotechnol..

[16] Zhao X., Wang H., Bo L., Zhao H., Li L., Zhou Y. (2018). Serum Lipid Level and Lifestyles Are Associated with Carotid Femoral Pulse Wave Velocity among Adults: 4.4-Year Prospectively Longitudinal Follow-Up of a Clinical Trial. Clin. Exp. Hypertens..

[17] Tomiyama H., Shiina K. (2020). State of the Art Review: Brachial-Ankle PWV. J. Atheroscler. Thromb..

[18] Bramwell J., Hill A. (1922). Velocity of Transmission of the Pulse-Wave: And Elasticity of Arteries. Lancet.

[19] Mancia G., Fagard R., Narkiewicz K., Redon J., Zanchetti A., Böhm M., Christiaens T., Cifkova R., De Backer G., Dominiczak A. (2013). 2013 ESH/ESC Guidelines for the Management of Arterial Hypertension: The Task Force for the Management of Arterial Hypertension of the European Society of Hypertension (ESH) and of the European Society of Cardiology (ESC). Eur. Heart J..

[20] Brands P.J., Willigers J.M., Ledoux L.A., Reneman R.S., Hoeks A.P. (1998). A Noninvasive Method to Estimate Pulse Wave Velocity in Arteries Locally by Means of Ultrasound. Ultrasound Med. Biol..

[21] Rabben S.I., Stergiopulos N., Hellevik L.R., Smiseth O.A., Slørdahl S., Urheim S., Angelsen B. (2004). An Ultrasound-Based Method for Determining Pulse Wave Velocity in Superficial Arteries. J. Biomech..

[22] Hsu Y.-P., Young D.J. (2014). Skin-Coupled Personal Wearable Ambulatory Pulse Wave Velocity Monitoring System Using Microelectromechanical Sensors. IEEE Sens. J..

[23] Pereira T., Correia C., Cardoso J. (2015). Novel Methods for Pulse Wave Velocity Measurement. J. Med. Biol. Eng..

[24] Munakata M. (2016). Brachial-Ankle Pulse Wave Velocity: Background, Method, and Clinical Evidence. Pulse.

[25] Sugawara J., Hayashi K., Tanaka H. (2014). Arterial Path Length Estimation on Brachial-Ankle Pulse Wave Velocity: Validity of Height-Based Formulas. J. Hypertens..

[26] Nabeel P., Karthik S., Joseph J., Sivaprakasam M. (2018). Arterial Blood Pressure Estimation from Local Pulse Wave Velocity Using Dual-Element Photoplethysmograph Probe. IEEE Trans. Instrum. Meas..

[27] Katsuda S.-I., Takazawa K., Miyake M., Kobayashi D., Kusanagi M., Hazama A. (2014). Local Pulse Wave Velocity Directly Reflects Increased Arterial Stiffness in a Restricted Aortic Region with Progression of Atherosclerotic Lesions. Hypertens. Res..

[28] Nabeel P.M., Joseph J., Sivaprakasam M. (2017). A Magnetic Plethysmograph Probe for Local Pulse Wave Velocity Measurement. IEEE Trans. Biomed. Circuits Syst..

[29] Huang J.J., Huang Y.M., Chang M.W. Using Bioimpedance Plethysmography for Measuring the Pulse Wave Velocity of Peripheral Vascular. Proceedings of the 2016 13th International Conference on Electrical Engineering/Electronics, Computer, Telecommunications and Information Technology (ECTI-CON).

[30] Joly L., Perret-Guillaume C., Kearney-Schwartz A., Salvi P., Mandry D., Marie P.-Y., Karcher G., Rossignol P., Zannad F., Benetos A. (2009). Pulse Wave Velocity Assessment by External Noninvasive Devices and Phase-Contrast Magnetic Resonance Imaging in the Obese. Hypertension.

[31] Hoctor R.T., Dentinger A.M., Thomenius K.E. Signal Processing for Ultrasound-Based Arterial Pulse Wave Velocity Estimation. Proceedings of the IEEE Ultrasonics Symposium.

[32] Runciman J., McGregor M., Silva G., Monteith G., Viel L., Arroyo L.G. (2016). A New Statistical Phase Offset Technique for the Calculation of In Vivo Pulse Wave Velocity. Artery Res..

[33] Sondej T., Sieczkowski K., Olszewski R., Dobrowolski A. (2019). Simultaneous Multi-Site Measurement System for the Assessment of Pulse Wave Delays. Biocybern. Biomed. Eng..

[34] Huttunen J.M.J., Kärkkäinen L., Lindholm H. (2019). Pulse Transit Time Estimation of Aortic Pulse Wave Velocity and Blood Pressure Using Machine Learning and Simulated Training Data. PLoS Comput. Biol..

[35] Sieczkowski K., Sondej T., Dobrowolski A., Olszewski R. (2016). Autocorrelation Algorithm for Determining A Pulse Wave Delay. Proceedings of the 2016 Signal Processing: Algorithms, Architectures, Arrangements, and Applications (SPA).

[36] Wang J., Zhu Y., Wu Z., Zhang Y., Lin J., Chen T., Liu H., Wang F., Sun L. (2022). Wearable Multichannel Pulse Condition Monitoring System Based on Flexible Pressure Sensor Arrays. Microsyst. Nanoeng..

[37] Tang Z., Wang S., Shi C. (2021). Development of a Hybrid Force-Displacement Sensor Based on Fiber Bragg Grating for Radial Artery Pulse Waveform Measurement. IEEE Sens. J..

[38] Padma S., Umesh S., Srinivas T., Asokan S. (2018). Carotid Arterial Pulse Waveform Measurements Using Fiber Bragg Grating Pulse Probe. IEEE J. Biomed. Health Inform..

[39] Li S.-J., Zhang F.-X., Ni J.-S., Wang C. Design of Pulse Pressure Sensor Based on Fiber Bragg Grating and Its Application in the Measurement of Pulse Condition Information. Proceedings of the 2016 IEEE International Conference on Information and Automation (ICIA).

[40] Massaroni C., Zaltieri M., Presti D.L., Nicolo A., Tosi D., Schena E. (2021). Fiber Bragg Grating Sensors for Cardiorespiratory Monitoring: A Review. IEEE Sens. J..

[41] Sharath U., Sukreet R., Apoorva G., Asokan S. (2013). Blood Pressure Evaluation Using Sphygmomanometry Assisted by Arterial Pulse Waveform Detection by Fiber Bragg Grating Pulse Device. J. Biomed. Opt..

[42] Sharath U., Shwetha C., Anand K., Asokan S. (2014). Radial Arterial Compliance Measurement by Fiber Bragg Grating Pulse Recorder. J. Hum. Hypertens..

[43] Sharath U., Goggi S.P., Ambastha S., Kalegowda A., Asokan S. (2015). Pulse Transit Time Differential Measurement by Fiber Bragg Grating Pulse Recorder. J. Biomed. Opt..

[44] Obeid H., Fortier C., Garneau C.-A., Pare M., Boutouyrie P., Bruno R.M., Khettab H., Goupil R., Agharazii M. (2021). Radial-Digital Pulse Wave Velocity: A Noninvasive Method for Assessing Stiffness of Small Conduit Arteries. Am. J. Physiol. Circ. Physiol..

[45] Chen W., Kobayashi T., Ichikawa S., Takeuchi Y., Togawa T. (2000). Continuous Estimation of Systolic Blood Pressure Using the Pulse Arrival Time and Intermittent Calibration. Med. Biol. Eng. Comput..

[46] McCombie D.B., Shaltis P.A., Reisner A.T., Asada H.H. Adaptive Hydrostatic Blood Pressure Calibration: Development of a wearable, Autonomous Pulse Wave Velocity Blood Pressure Monitor. Proceedings of the 2007 29th Annual International Conference of the IEEE Engineering in Medicine and Biology Society.

[47] Schäfer A., Vagedes J. (2013). How Accurate Is Pulse Rate Variability as an Estimate of Heart Rate Variability? A Review on Studies Comparing PhotoplethysmoGraphic Technology with an Electrocardiogram. Int. J. Cardiol..

